# Development of a nomogram for predicting malnutrition in elderly hospitalized cancer patients: a cross-sectional study in China

**DOI:** 10.3389/fnut.2024.1396293

**Published:** 2024-07-08

**Authors:** Ran Duan, Yan Luo, Tong Feng, Tao Ren

**Affiliations:** ^1^Clinical Medical College, Chengdu Medical College, Chengdu, Sichuan, China; ^2^Department of Oncology, Clinical Medical College and The First Affiliated Hospital of Chengdu Medical College, Chengdu, China; ^3^Department of Respiratory and Critical Care Medicine, The Second People’s Hospital of Xindu District, Chengdu, China; ^4^The Second School of Clinical Medicine, Southern Medical University, Guangzhou, China; ^5^Department of Oncology, The First Affiliated Hospital of Traditional Chinese Medical of Chengdu Medical College·Xindu Hospital of Traditional Chinese Medica, Chengdu, China; ^6^Radiology and Therapy Clinical Medical Research Center of Sichuan Province, Chengdu, China; ^7^Clinical Key Speciality (Oncology Department) of Sichuan Province, Chengdu, China

**Keywords:** malignant tumor, nomogram, malnutrition, prediction model, older

## Abstract

**Objectives:**

The Patient-Generated Subjective Global Assessment (PG-SGA) serves as a specialized nutritional assessment instrument designed for cancer patients. Despite its specificity, the complexity and time requirements of this tool, along with the necessity for administration by trained professionals, limit its practicality in clinical settings. Our objective is to identify a straightforward, efficient, and dependable nutritional assessment tool to promote broader adoption in clinical practice.

**Methods:**

This study encompassed a total of 450 patients diagnosed with cancer. Of these, 315 individuals constituted the training set, and the remaining 135 were allocated to the external validation set. The model variables were identified through the Least Absolute Shrinkage and Selection Operator (LASSO) regression method. Binary logistic regression outcomes facilitated the development of a nomogram, offering a visual depiction of the predicted probabilities. The predictive accuracy of the nomogram model was evaluated by calculating the area under the Receiver Operating Characteristic (ROC) curve.

**Results:**

The LASSO method detected four variables that were included in the final prediction model: age, serum albumin levels (ALB), body mass index (BMI), and activities of daily living (ADL). The area under the curve (AUC) for this prediction model was 0.905. Both the internal and external calibration curves for malnutrition showed that the predictive nomogram model was highly accurate.

**Conclusion:**

The study has developed a prediction model that demonstrates remarkable accuracy in forecasting malnutrition. Furthermore, it presents a streamlined nutritional assessment tool aimed at swiftly identifying cancer patients at nutritional risk, thereby facilitating oncologists in delivering targeted nutritional support to these individuals.

## Introduction

Cancer patients are highly susceptible to malnutrition, which is implicated in at least 20% of mortalities within this group (1). Malnutrition compromises the tolerance of patients to radiotherapy and chemotherapy, hindering their ability to adhere to prescribed treatment schedules. This interference not only affects the efficacy of treatments but also results in extended hospitalizations and elevated mortality rates (2, 3). Consequently, the provision of tailored nutritional support, initiated with a comprehensive nutritional assessment, is imperative for improving patient outcomes (4). By leveraging predictive models, targeted nutritional interventions can substantially enhance the nutritional well-being of patients, mitigate the risk of treatment-related complications, and elevate their quality of life.

The Patient-Generated Subjective Global Assessment (PG-SGA), adapted for the specific needs of cancer patients, offers a nuanced approach to nutritional evaluation (5). It employs a continuous scoring mechanism that facilitates the early detection of subtle nutritional shifts and supports the ongoing monitoring of nutritional status through periodic reviews. Nonetheless, the intricate and labor-intensive nature of this assessment, compounded by its dependence on subjective evaluations from both patients and healthcare providers, introduces potential biases in the screening process (6). The effective implementation of the PG-SGA necessitates the involvement of medical personnel with specialized training, presenting a formidable barrier in resource-constrained settings.

Tian and his team conducted a retrospective study on clinical data from 344 patients with gastric cancer who underwent laparoscopic surgery, dividing the data into a training set and a validation set in a 7:3 ratio (7). They then developed a nutritional risk assessment model using logistic regression for patients post-gastrectomy. In another study, Yin et al. (8) analyzed data from 1,219 lung cancer patients in a multicenter observational cohort study, creating a prediction model with an AUC of 0.982 through traditional logistic regression that included variables such as gender, body mass index, weight loss over 6 months, leg circumference, and hand grip strength ratio. However, this model focused solely on a single type of cancer and lacked a specific nutritional risk screening tool for elderly cancer patients. Yu et al. (9) collected X-ray computed tomography (CT) scans from 120 cervical cancer patients prior to radiotherapy and chemotherapy. They analyzed non-enhanced CT images to determine the radiological characteristics of the L3 psoas major muscle, using LASSO regression for predicting malnutrition in the dataset, though the data were challenging to collect and had limited clinical utility. Meanwhile, Zhang et al. (10) retrospectively analyzed medical records from 702 cancer patients, selecting age, left arm phase angle, and BMI as predictors to construct decision tree and random forest models. The models showed robust performance with an AUC of 0.813, but retrospective study limitations prevented the collection of potentially relevant information such as smoking, drinking status, and education level. Patients with different types of cancer may face multiple risk factors simultaneously, with each factor contributing variably to the development of malnutrition. Based on the identification of nutritional risk factors in elderly cancer patients, it is worth investigating whether a small number of easily collected and cost-effective indicators can accurately predict the risk of malnutrition.

Utilizing sophisticated mathematical formulas, a risk prediction model is designed to precisely calculate the probability that an individual is presently dealing with a specific disease or will likely face a particular health outcome in the foreseeable future (11, 12). This research aims to develop a straightforward, swift, and dependable nutritional assessment tool specifically for the elderly cancer patient population, culminating in the creation of a user-friendly nomogram to facilitate widespread clinical adoption.

## Materials and methods

### Patients and study design

**Figure 1:**
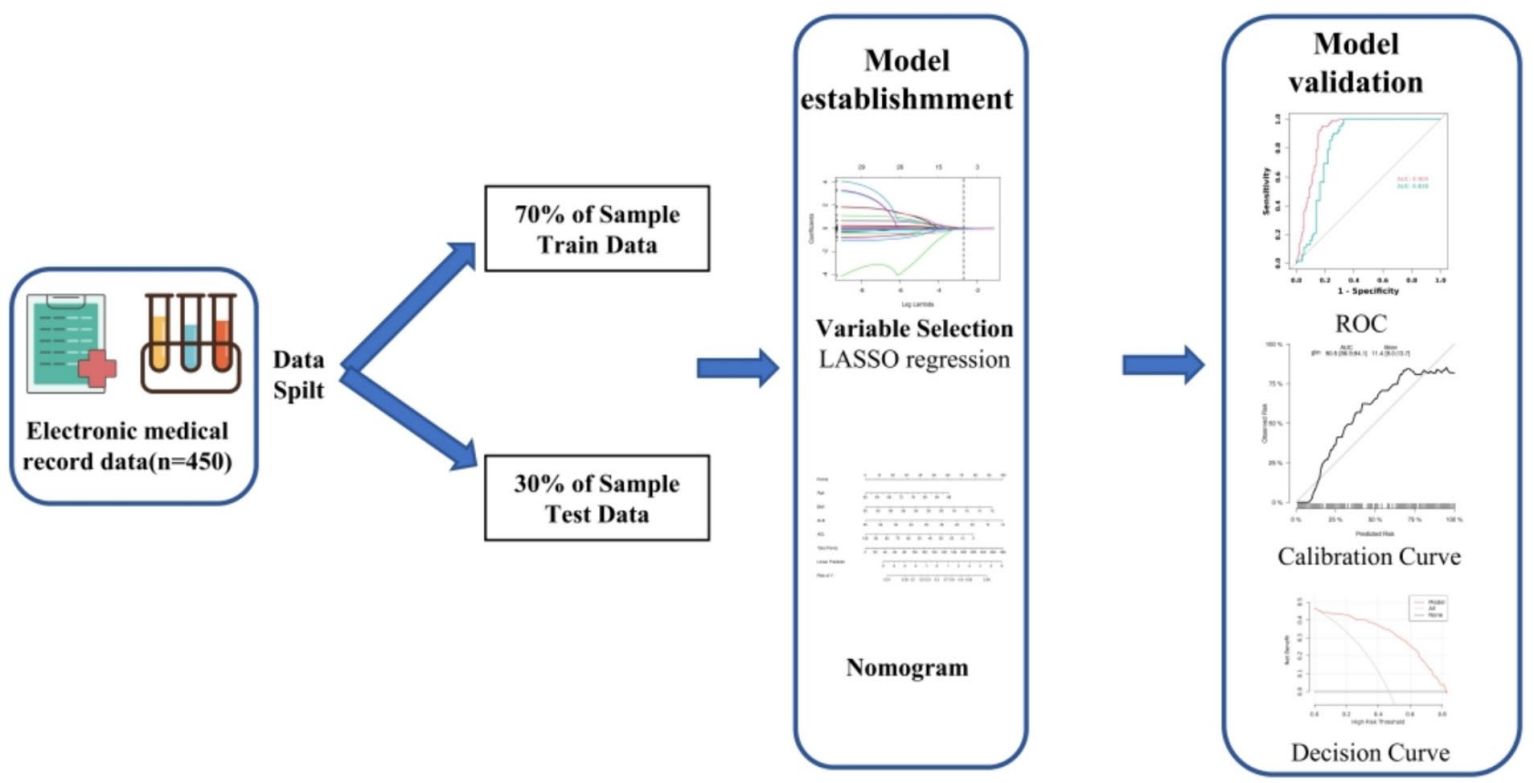
The overview of study design.

**Figure 2:**
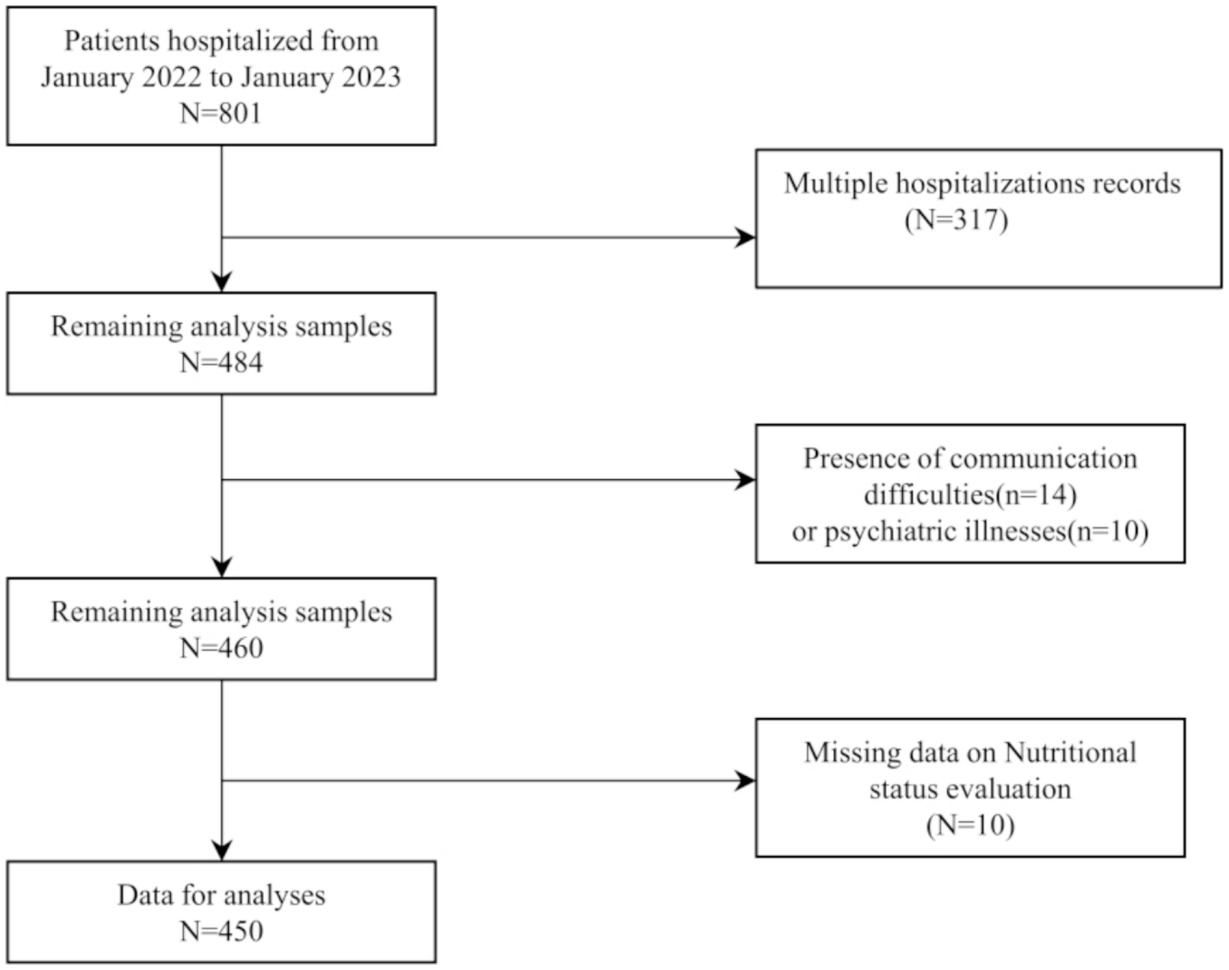
Flowchart of participant selection.

The study selected elderly patients hospitalized in the Oncology Department of the First Affiliated Hospital of Chengdu Medical College from January 2022 to January 2023. To qualify for the study, participants were required to meet certain criteria. They needed to be at least 60 years old and diagnosed with cancer. Exclusion criteria included patients with repeated hospital admissions, communication barriers, psychiatric conditions, or incomplete nutritional status data. The overview of the study can be seen in Figure 1, and the process of participant recruitment and enrollment is depicted in Figure 2 of the research report.

The study was carried out in accordance with the relevant guidelines and regulations of Declaration of Helsinki and was approved by the Ethics Committee of The First Affiliated Hospital of Chengdu Medical College (2024CYFIRB-BA-Mar 09). The data are anonymous, and the requirement for informed consent was therefore waived.

### Data collection

In this study, a total of 22 socio-demographic and clinical parameters were collected for analysis. These parameters span a variety of aspects, including (1) demographic characteristics such as age, gender, body mass index, and education level; (2) lifestyle behaviors, specifically alcohol consumption and smoking; and (3) history and indicators related to malnutrition, including diabetes, hypertension, pain scores, cancer staging, chemotherapy status, radiotherapy status, immunotherapy status, and various biochemical indices like albumin, globulin, leukocytes, neutrophils, and C-reactive protein. The ability to perform activities of daily living (ADL) was assessed using the Barthel Index, which evaluates 10 items including eating, dressing, bathing, bowel control, bladder control, grooming, moving up and down stairs, transferring, using the toilet, and walking. The total score ranges from 0 to 100, with scores above 60 indicating basic self-sufficiency in daily activities and scores below 60 indicating the need for partial assistance (13).

### Definition and assessment

The nutritional status of the selected patients was evaluated using the Patient-Generated Subjective Global Assessment (PG-SGA) questionnaire. The PG-SGA questionnaire consists of two parts: the PG section, which is completed by the patient through self-assessment and includes information on recent weight changes, recent dietary intake, symptoms and signs related to diet, activity, and function; and the SGA section, which is filled out by the investigator after assessment, including the patient’s age at onset of disease, metabolic stress status, and physical examination findings. The total score from both sections constitutes the PG-SGA score, with higher scores indicating worse nutritional status. A PG-SGA score of 4 or above is indicative of malnutrition (14).

### Statistical analysis

According to the principle of (15), in a regression model, at least 10 events per variable (EPV) outcome events are required for each included variable. In this study, the outcome events refer to cancer patients experiencing malnutrition. According to the type of missing values, random forest filling method is used to deal with the missing data. With four variables included in the model, 40 outcome events need to be included, indicating the presence of 40 patients experiencing malnutrition. To construct and validate the nomogram, participants were divided into a training set and a validation set randomly, with their comparability subsequently assessed. Continuous variables were analyzed using the Wilcoxon rank-sum test and reported as mean ± standard deviation. Categorical data were expressed as numbers (percentage) and compared using the chi-square test.

LASSO regression was primarily utilized in our study for its robust feature selection capabilities, particularly useful given the large set of predictors initially considered. This approach efficiently reduced multicollinearity and enhanced the interpretability of our model by selecting the most relevant features without overfitting. This was followed by multivariate logistic regression analysis to construct the nomograms. Subjects were randomly allocated to the training and validation sets in a 7:3 ratio, with the training set used to build the predictive model. The predictive accuracy of the nomograms was evaluated through the area under the curve (AUC) of the receiver operating characteristic (ROC) curve in both sets (16). Calibration curves assessed the concordance between actual outcomes and predicted probabilities, while the nomograms’ clinical utility was determined using decision curve analysis (DCA) (17). All statistical analyses were conducted using R software version 4.2, with a *p*-value of less than 0.05 deemed statistically significant for all tests.

## Results

### Characteristics of subjects

In this study, the incidence of malnutrition was observed at 46.4%, affecting 209 of the 450 participants involved. Further examination of the data revealed that within the training group, 147 out of 315 participants (46.67%) experienced malnutrition, whereas in the validation group, 62 out of 135 participants (45.92%) were found to have the condition. Table 1 provides a detailed summary of the demographic and clinical characteristics of the participants. It is important to note that there were no significant differences among the participants regarding gender, education level, radiotherapy, chemotherapy, immunotherapy, smoking status, hypertension, hemoglobin levels, globulin levels, neutrophil, and leukocyte counts.

**Table 1:** Baseline characteristics of the training set and validation set.

Characteristics	Training cohort			Internal test cohort		
	0, *N* = 168^1^	1, *N* = 147^1^	*p*-value^2^	0, *N* = 73^1^	1, *N* = 62^1^	*p*-value^2^
Gender, *N* (%)			0.265			0.989
Male	103 (61%)	99 (67%)		46 (63%)	39 (63%)	
Female	65 (39%)	48 (33%)		27 (37%)	23 (37%)	
Age			<0.001			0.022
Mean ± *SD*	68 ± 6	73 ± 6		68.3 ± 5.2	70.6 ± 6.3	
Education, *N* (%)			0.508			0.374
Below High School	149 (90%)	137 (93%)		67 (92%)	60 (97%)	
High School	7 (4%)	5 (3%)		1 (1%)	1 (2%)	
Above High school	10 (6%)	5 (3%)		5 (7%)	1 (2%)	
Radiotherapy, *N* (%)			0.067			0.973
No	115 (68%)	86 (59%)		41 (56%)	35 (56%)	
Yes	53 (32%)	61 (41%)		32 (44%)	27 (44%)	
Chemotherapy, *N* (%)			0.412			0.461
No	61 (36%)	60 (41%)		28 (38%)	20 (32%)	
Yes	107 (64%)	87 (59%)		45 (62%)	42 (68%)	
Immunotherapy, *N* (%)			0.338			0.145
No	152 (90%)	128 (87%)		68 (93%)	53 (85%)	
Yes	16 (10%)	19 (13%)		5 (7%)	9 (15%)	
Pain score			0.030			0.004
Mean ± *SD*	0.21 ± 0.64	0.38 ± 0.71		0.25 ± 0.68	0.74 ± 1.16	
T			0.019			0.552
0	2 (1%)	1 (1%)		0 (0%)	0 (0%)	
1	10 (6%)	0 (0%)		5 (7%)	1 (2%)	
2	20 (12%)	23 (16%)		5 (7%)	5 (8%)	
3	76 (45%)	68 (46%)		37 (51%)	31 (50%)	
X	60 (36%)	55 (37%)		26 (36%)	25 (40%)	
N			0.643			0.364
0	23 (14%)	13 (9%)		6 (8%)	4 (6%)	
1	26 (15%)	23 (16%)		8 (11%)	10 (16%)	
2	61 (36%)	56 (38%)		35 (48%)	21 (34%)	
3	15 (9%)	18 (12%)		6 (8%)	4 (6%)	
X	43 (26%)	37 (25%)		18 (25%)	23 (37%)	
M			0.710			0.123
0	105 (63%)	90 (61%)		50 (68%)	32 (52%)	
1	52 (31%)	50 (34%)		20 (27%)	27 (44%)	
X	11 (7%)	7 (5%)		3 (4%)	3 (5%)	
Smoke, *N* (%)			0.594			0.202
No	58 (35%)	55 (37%)		24 (33%)	27 (44%)	
Yes	110 (65%)	92 (63%)		49 (67%)	35 (56%)	
Diabetes, *N* (%)			<0.001			0.986
No	159 (95%)	117 (80%)		66 (90%)	56 (90%)	
Yes	9 (5%)	30 (20%)		7 (10%)	6 (10%)	
Drink, *N* (%)			<0.001			0.920
No	10 (6%)	28 (19%)		9 (12%)	8 (13%)	
Yes	158 (94%)	119 (81%)		64 (88%)	54 (87%)	
Hypertension, *N* (%)			0.112			0.992
No	134 (80%)	106 (72%)		60 (82%)	51 (82%)	
Yes	34 (20%)	41 (28%)		13 (18%)	11 (18%)	
BMI			<0.001			<0.001
Mean ± *SD*	22.09 ± 3.05	19.34 ± 2.79		21.40 ± 2.93	18.84 ± 2.74	
Hemoglobin			0.575			<0.001
Mean ± *SD*	115 ± 17	114 ± 37		121 ± 21	107 ± 22	
Globulin			0.149			0.956
Mean ± *SD*	28.6 ± 4.9	29.4 ± 5.4		29.6 ± 6.3	29.6 ± 5.2	
C-Reactive Protein			<0.001			0.010
Mean ± *SD*	14 ± 17	32 ± 36		24 ± 37	40 ± 37	
Neutrophils			0.153			0.997
Mean ± *SD*	5.49 ± 9.91	4.34 ± 2.76		6.0 ± 8.6	6.0 ± 9.0	
ALB			<0.001			<0.001
Mean ± *SD*	39.7 ± 4.3	35.7 ± 6.4		40 ± 6	34 ± 6	
Leukocyte			0.765			0.527
Mean ± *SD*	5.78 ± 3.00	5.68 ± 2.89		6.7 ± 4.2	6.3 ± 3.4	
ADL			<0.001			<0.001
Mean ± SD	86 ± 28	39 ± 28		78 ± 33	43 ± 27	

^1^*n* (%). ^2^Pearson’s Chi-squared test; Welch Two Sample *t*-test; Fisher’s exact test. BMI, body mass index; ALB, albumin; ADL, activities of daily living.

### Construction of nomogram

**Figure 3:**
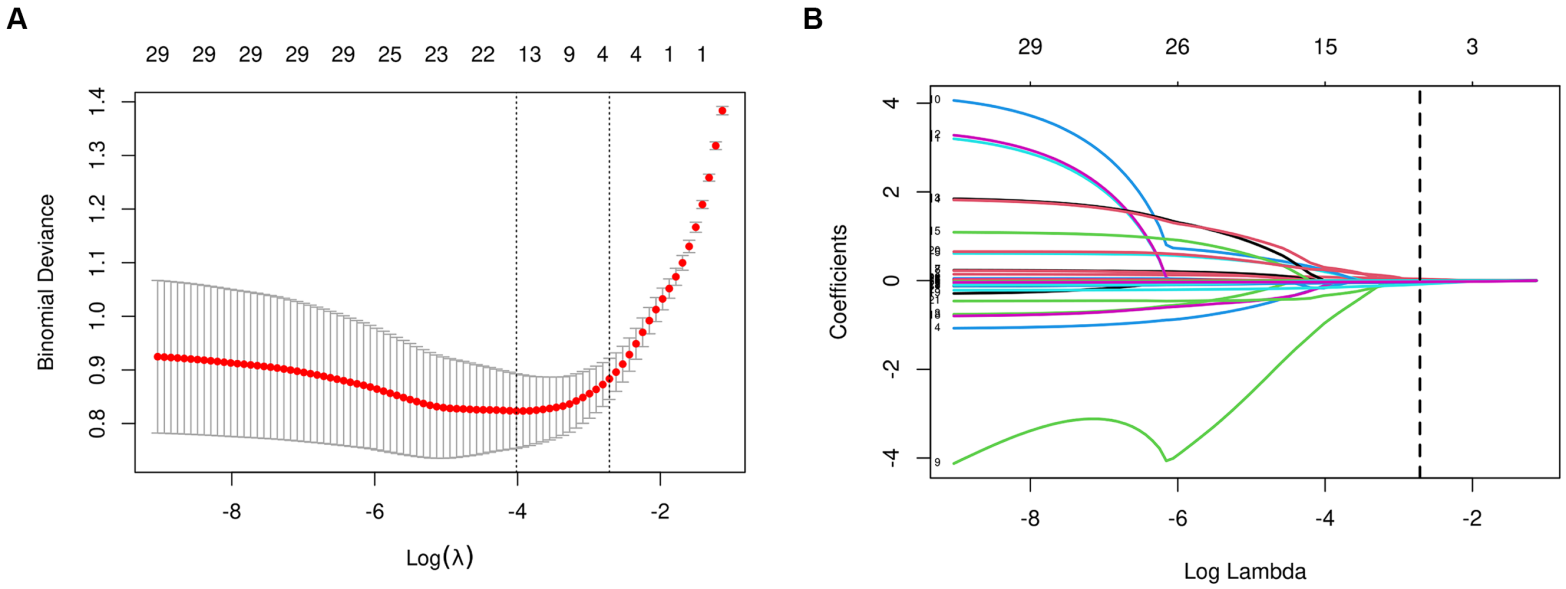
Features selection used the LASSO regression. **(A)** The tuning parameter (lambda) selection is based on the deviance in the LASSO regression, using both the minimum criteria (indicated by the left dotted line) and the 1-SE criteria (indicated by the right dotted line). **(B)** A coefficient profile plot is created against the logarithmic (lambda) sequence. In this study, the selection of predictors is based on the minimum criteria (indicated by the right dotted line), resulting in the selection of four non-zero coefficients using the LASSO regression model. LASSO, least absolute shrinkage and selection operator; SE, standard error.

**Figure 4:**
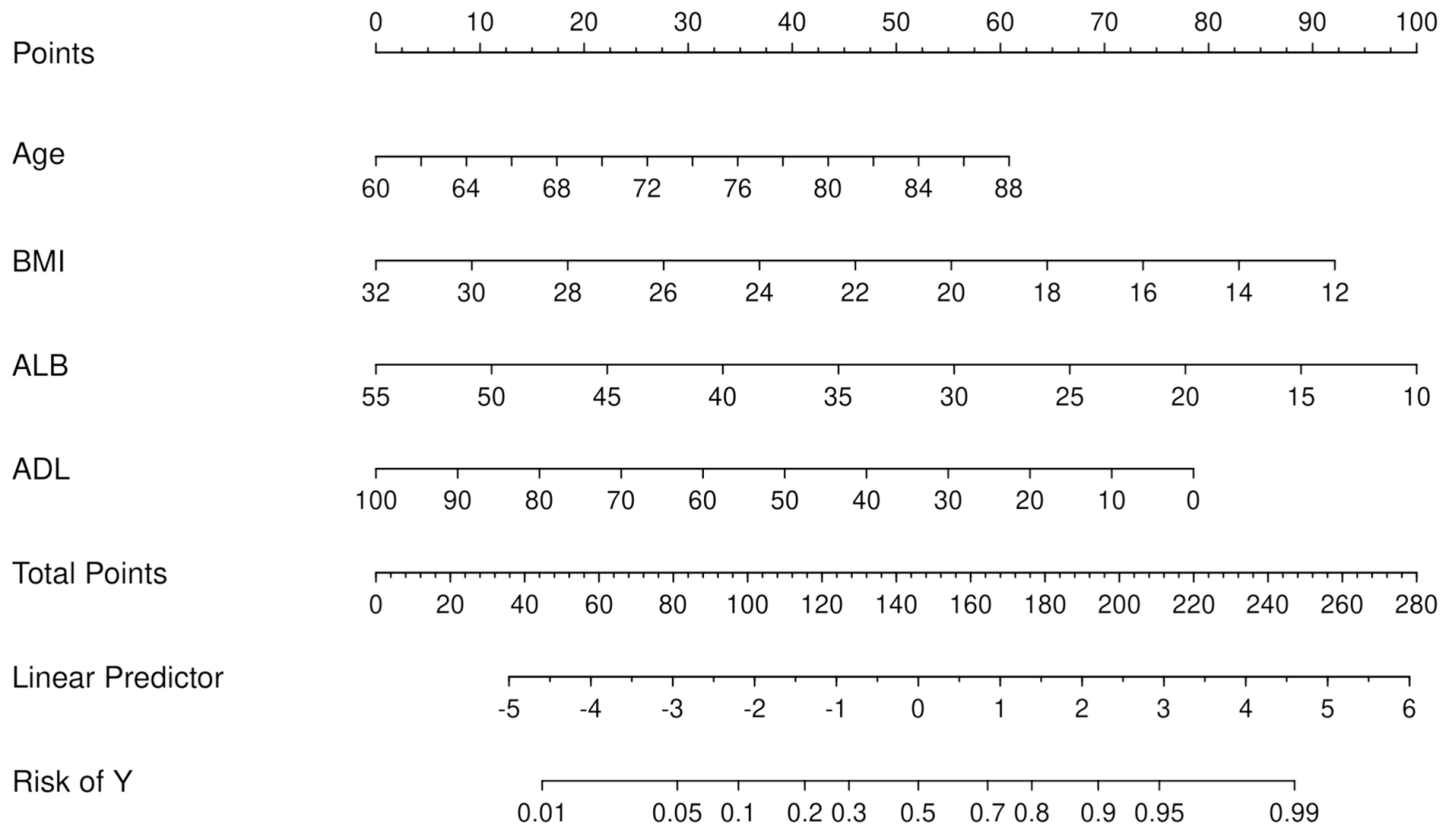
Nomogram for the prediction of malnutrition. First, a score was assigned to each variable. These scores were then summed to obtain a total point count. Finally, the corresponding predicted probability of malnutrition was determined based on the lowest category.

From the 22 variables collected, LASSO regression analysis identified 4 variables with non-zero coefficients for selection, as depicted in Figure 3. The coefficients from the LASSO regression analysis are detailed in Supplementary Table S1. The variables selected were ADL, ALB, BMI, and age. The relationship between each individual factor and the outcome of malnutrition, along with the ROC curve, can be found in Supplementary Table S2 and Supplementary Figure 1. A multivariate logistic regression analysis incorporating these 4 factors was conducted to develop the nomogram, which is presented in Table 2 and Figure 4.

**Table 2:** Multivariable logistic regression analysis based on the four variables selected by the LASSO regression technique.

Characteristic	*N*	Event *N*	OR^1^	95% CI^1^	*p*-value
Age	315	147	1.10	1.05, 1.16	<0.001
BMI	315	147	0.81	0.72, 0.91	<0.001
ALB	315	147	0.90	0.85, 0.96	0.002
ADL	315	147	0.96	0.96, 0.97	<0.001

^1^OR, Odds Ratio; CI, Confidence Interval. BMI, body mass index; ALB, albumin; ADL, activities of daily living.

### Assessment of nomogram in the training set and validation set

**Figure 5:**
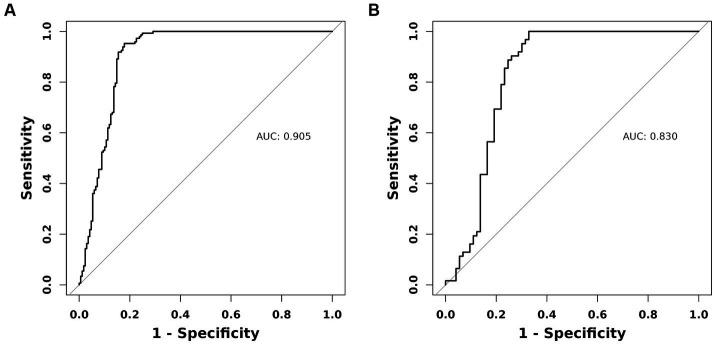
The AUC (representative the discriminatory ability of the model) of the training set and the validation set. **(A)** Training set. **(B)** Validation set. AUC, area under the curve.

**Figure 6:**
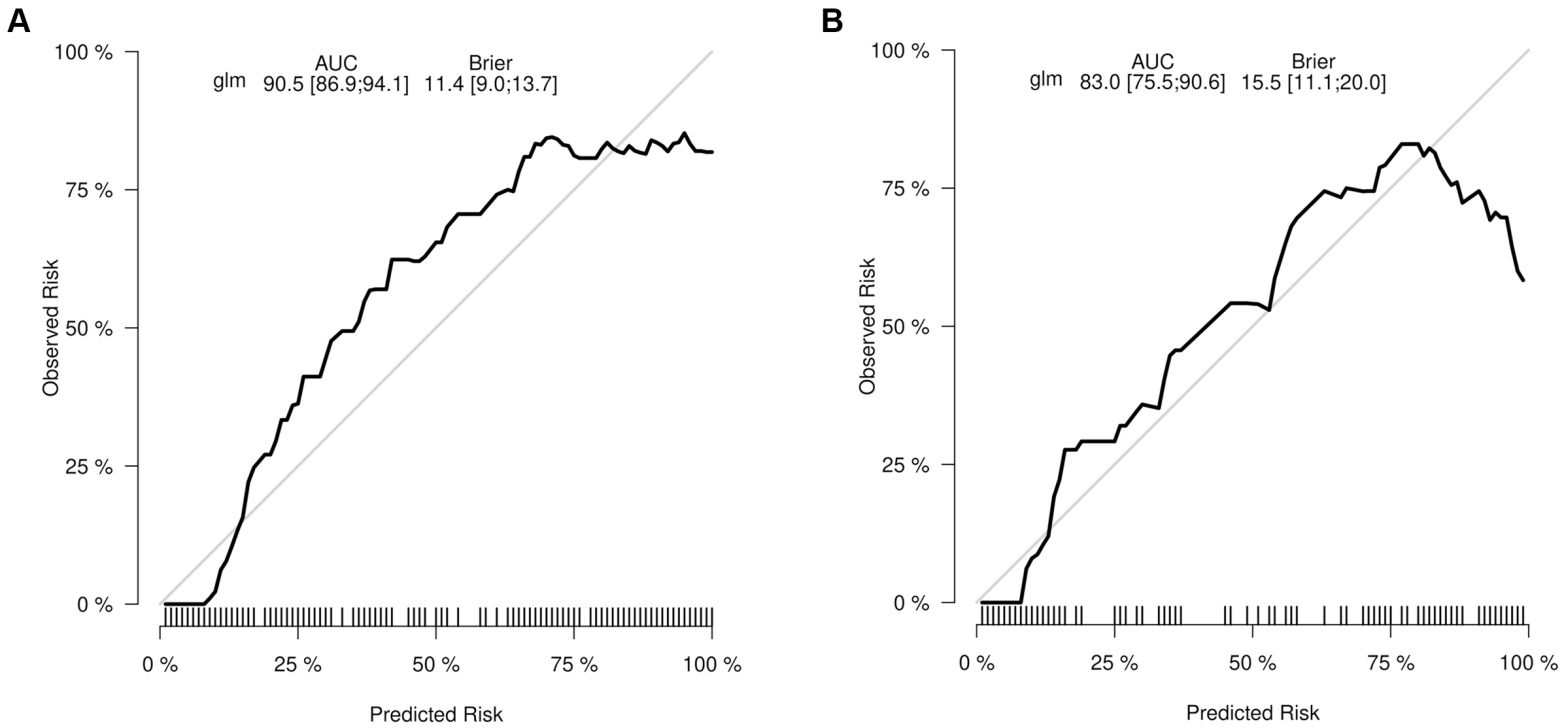
The calibration curve depicts the extent of consistency between the predicted probability and observed probability. The *y*-axis represents the actual probability of malnutrition, while the *x*-axis represents the nomogram-predicted probability of malnutrition. The presence of a diagonal line signifies that the predicted probability is equal to the actual probability. Any deviations from this diagonal line indicate the magnitude of prediction error. **(A)** Training set. **(B)** Validation set.

As previously mentioned, the nomogram was specifically developed to determine the likelihood of developing malnutrition by taking into account various variables such as ADL, ALB, BMI, and age. To assess the performance of the nomogram, the AUC was utilized as a measure. The AUC value obtained for the nomogram was 0.905 as depicted in Figure 5A. Furthermore, the calibration curve manifested a significant level of agreement between the predicted probabilities and the actual outcomes, as illustrated by Figure 6A. In this validation set, the AUC obtained for the nomogram was 0.830 as shown in Figure 5B. Moreover, the calibration curve of this set demonstrated a high level of consistency between the predicted probabilities and the observed outcomes, as depicted in Figure 6B.

### Clinical utility of nomogram

**Figure 7:**
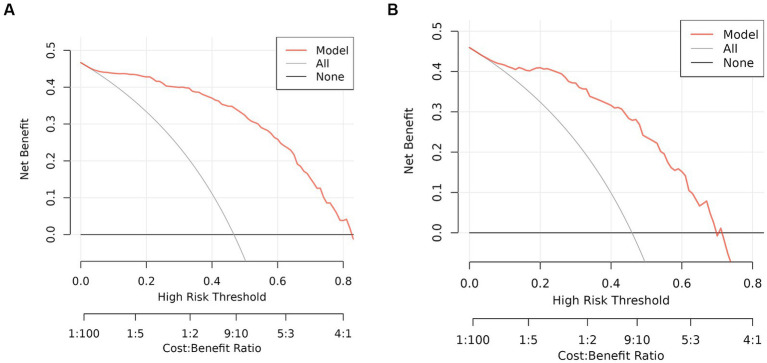
DCA of the nomogram. **(A)** Training set. **(B)** Validation set. DCA, Decision curve analysis.

The DCA results demonstrate that utilizing this nomogram in our current study to predict the risk of malnutrition could provide a greater benefit within a threshold probability range of 5–82% (Figure 7A). These findings were confirmed in the validation set, where the threshold probability range was 9–70% (Figure 7B).

### Website of nomogram

**Figure 8:**
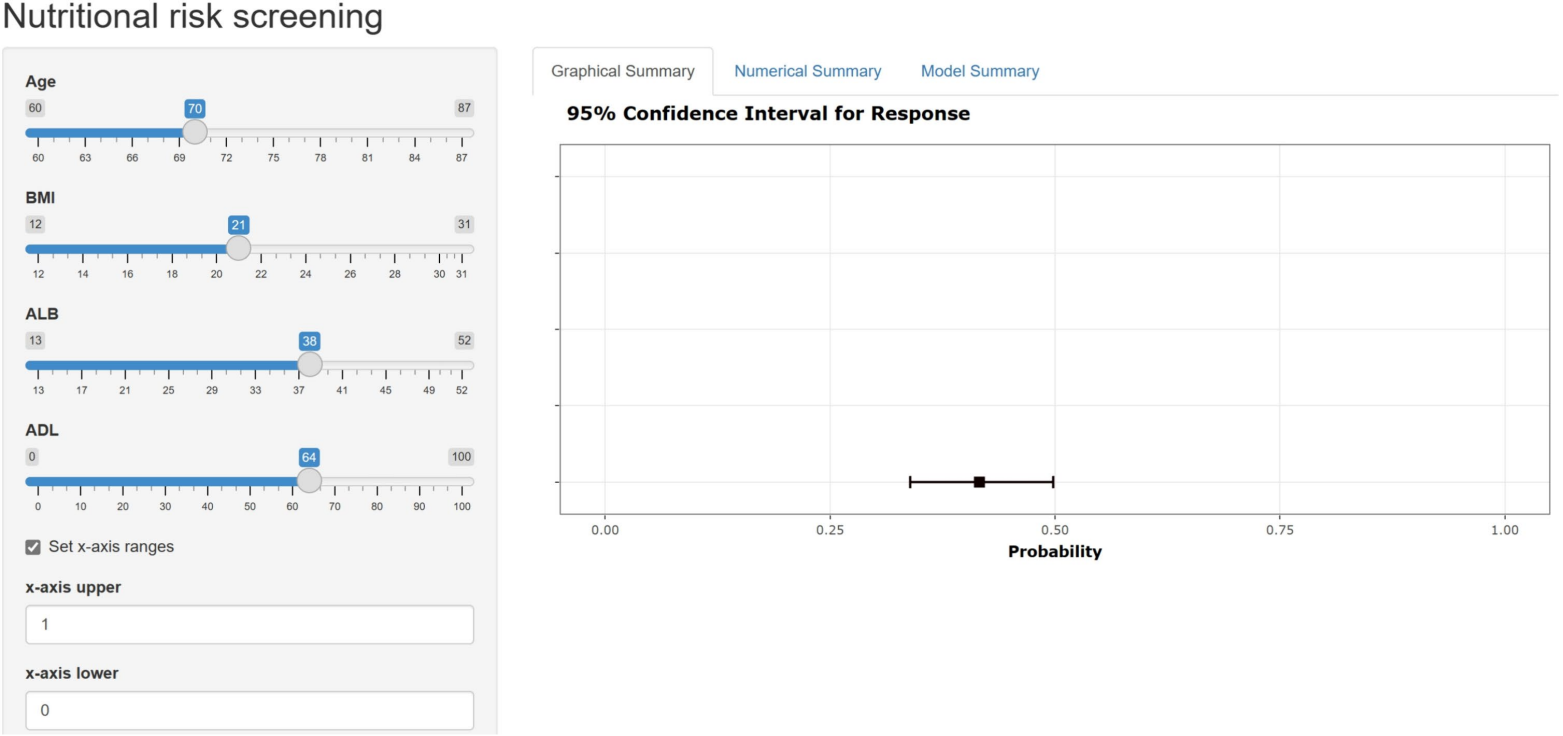
The web-based user-friendly calculator of nomogram (https://duanran.shinyapps.io/dynnomapp/).

A user-friendly web-based tool, known as a nomogram calculator, has been created and is readily accessible online for free (Figure 8). This calculator has been specifically designed with the aim of aiding both patients and healthcare professionals in estimating the likelihood of developing malnutrition.

## Discussion

We developed a statistical model to predict malnutrition in elderly cancer inpatients. The nomogram, grounded in common demographic and laboratory biochemical markers, demonstrates satisfactory discrimination and calibration. The clinical utility of this nomogram, as evidenced by DCA, confirms its efficacy in predicting malnutrition among cancer patients. Additionally, we have created an online risk calculator that accurately estimates the likelihood of malnutrition in cancer patients, aiding healthcare professionals in devising targeted diagnostic and treatment strategies for malnutrition screening.

Tian and other researchers retrospectively collected the clinical data of 344 patients with gastric cancer who underwent laparoscopic surgery, and divided the data into a training set and a validation set in a 7:3 ratio (7). Using logistic regression, they established a nutritional risk assessment model for patients with gastric cancer post-gastrectomy. In a multicenter, observational cohort study, Yin et al. (8) analyzed the data of 1,219 patients with lung cancer. They utilized the traditional logistic regression method to construct a prediction model containing six variables: gender, body mass index, weight loss within 6 months, weight loss after 6 months, leg circumference, and the ratio of hand grip strength to body weight. The AUC value of the model was 0.982 (95% confidence interval, 0.969–0.995). Compared with these studies, this study enhanced the universality and practical value of the model by integrating data from various types of cancer, providing a feasible nutritional risk assessment tool for patients with different types of cancer. Additionally, through the use of LASSO regression, this research effectively addresses the issue of feature selection in high-dimensional data and can identify the most influential factors from a large number of potential predictive variables. This is crucial for clinicians to rapidly and accurately assess the risk of malnutrition in elderly cancer patients in a complex medical environment. Yu et al. (9) collected the X-ray computed tomography (CT) scanning data of 120 patients with cervical cancer before receiving radiotherapy and chemotherapy. By analyzing the non-enhanced CT images, they obtained the radiological characteristics of the L3 psoas major muscle. The research team used the Least Absolute Shrinkage and Selection Operator (LASSO) to predict malnutrition in the training dataset. In contrast, the indicators contained in our models are non-invasive and cost-effective, and data can be obtained through simple surveys and basic measurements at the time of admission. Zhang et al. (10) retrospectively analyzed the medical records of 702 cancer patients, selecting age, left arm phase angle, and BMI as predictive factors, and constructed a decision tree and random forest artificial neural network model. The results showed that the model performed well, with an AUC of 0.813, sensitivity of 75.9%, and specificity of 73.3%, and the actual survival curve was basically consistent with the prediction. However, due to the limitations of retrospective studies, the researchers failed to collect information such as smoking, drinking status, and education level. Our study includes these variables, which may help to improve the prediction ability of the model.

Malignant tumors lead to numerous severe complications, largely due to the tumor itself or inadequate treatment, significantly affecting patient outcomes and life quality. Resting Energy Expenditure (REE), the total energy consumed by the body at rest over a day, typically increases by approximately 10% in cancer patients (18). This increase demands greater food intake to meet bodily needs; failure to do so results in a negative energy balance and subsequent malnutrition. Malnutrition exacerbates cancer patients’ conditions, potentially leading to mortality, with malnutrition and its complications responsible for the death of about 20% of such patients (19). Various factors, including anorexia, nutrient metabolism disorders, altered energy consumption, and the impacts of radiotherapy, chemotherapy, and surgery, can induce malnutrition, either individually or in combination. Additionally, the incidence and mortality rates of malignant tumors correlate strongly with age, with the aging population in China contributing to a rapid increase in the tumor burden. Elderly cancer patients face a higher risk of nutritional deficiencies due to diminished gastrointestinal function and nutrient absorption efficiency, significant protein depletion by tumors, and the stress of surgical and chemotherapeutic interventions. Moreover, nutritional status is influenced by the tumor’s pathological type, disease duration, and progression, underscoring its pivotal role in tumor development and progression. Thus, maintaining adequate nutritional levels in patients is vital for enhancing quality of life and survival rates.

In our investigation, we identified a strong association between elevated ADL scores and a lower prevalence of malnutrition in the elderly population. The diminished capacity for conducting daily self-care activities emerges as a critical risk factor for malnutrition among older individuals, attributable to multiple factors: (1) Individuals with lower ADL scores experience reduced mobility, impairing their ability to access and prepare nutrient-rich foods (20); (2) A decline in self-care abilities results in decreased physical activity, reduced gastrointestinal motility, and compromised digestion and absorption functions, leading to nutritional deficiencies; (3) Moreover, impaired mobility can contribute to social isolation and diminished appetite, further elevating the risk of malnutrition (21).

The findings revealed a statistically significant correlation between serum ALB levels and malnutrition (OR = 0.90, 95% CI: 0.85–0.96, *p* < 0.001), suggesting that higher ALB levels are associated with a reduced risk of malnutrition. Ge et al. (22) corroborated these findings in their study on individuals with advanced lung cancer in Northern China. Serum albumin, produced by the liver, is pivotal for the transport of substances and nutritional support. Prior studies have confirmed a significant link between albumin levels and nutritional health, establishing it as a key biochemical marker for nutritional assessment. The influence of serum albumin on nutritional health stems from its roles in nutrient transport and nutrition; it binds to and transports various nutrients, including amino acids, fatty acids, and steroids (23, 24). Moreover, albumin is rich in essential amino acids such as leucine, threonine, and lysine, which, when metabolized, supply the building blocks for the production of other proteins, thereby facilitating protein synthesis and transformation within the body. A reduction in serum albumin levels can thus obstruct nutrient transport and conversion, leading to substantial nutritional deficits and heightened malnutrition risk (25). Additionally, serum albumin is critical for regulating blood’s colloidal osmotic pressure, with 75–80% of this pressure attributable to albumin. A decrease in albumin levels may result in edema across various body regions and organs. Specifically, gastrointestinal tract edema can impair nutrient absorption and digestion, further increasing the likelihood of malnutrition (26).

Contemporary research frequently employs nomograms to visualize prediction models. However, nomograms’ reliance on manual operations for observation and calculation presents notable limitations, particularly under conditions of medical resource scarcity, potentially hindering the effective implementation of malnutrition risk assessments. In response, this study introduces a web-based calculator, derived from the established nomogram, aimed at improving the clinical utility and dissemination of the malnutrition risk prediction model for lung cancer patients undergoing chemotherapy. By simply inputting patient data into specified webpage fields, healthcare professionals can quickly ascertain a patient’s malnutrition risk, streamlining the process. This calculator not only enhances computational efficiency and accuracy, markedly reducing the potential for human error associated with manual calculations but also optimizes healthcare workers’ productivity by eliminating cumbersome calculation steps.

In contrast to the conventional PG-SGA scale, our prediction model leverages objective metrics such as age, ALB, BMI, and ADL, expediting and simplifying the nutritional assessment process. This approach facilitates broader implementation of nutritional assessments in clinical settings and contributes to the standardization of oncology nutritional care. The model provides a valuable screening tool for clinicians and non-specialist healthcare providers to identify cancer patients at elevated risk of malnutrition, offering a quantitative risk assessment that aids in tailoring individualized treatment plans. Furthermore, it empowers nurses to conduct preventive health education for high-risk individuals, promoting nutritional awareness and emphasizing its critical role in patient and caregiver education. Such initiatives have the potential to mitigate the incidence of malnutrition during chemotherapy, thereby extending patient survival and enhancing quality of life.

Nonetheless, the study is not without its limitations. It is primarily based on a survey conducted in a single-center setting among elderly inpatients, which may introduce selection bias. Additionally, due to the retrospective nature of the study, it was not possible to collect information on surgeries, potentially limiting the predictive accuracy of our model. The relatively small sample size could also restrict the generalizability of our findings. Future research should therefore focus on multicenter, large-scale studies to externally validate and refine the prediction model. Incorporating advanced machine learning techniques into clinical evaluations could lead to more accurate and user-friendly tools for the early detection of malnutrition risk among elderly cancer inpatients, providing an invaluable resource for healthcare professionals. Considering the potential advantages of the GLIM criteria, we plan to conduct a subsequent analysis in our prospective study, reevaluating malnutrition under the GLIM criteria to offer more comprehensive guidance for clinical practice. We also intend to collaborate with other institutions across different regions to apply our model to a broader range of patient populations. This will include patients from varied ethnic backgrounds and healthcare settings, enabling us to evaluate how well our model performs under diverse demographic and clinical conditions.

## Conclusion

The study developed a prediction model incorporating four variables: age, ALB, BMI, and ADL. This model demonstrated significant predictive power for malnutrition. Its simplicity, non-invasive approach, and reliance on easily accessible variables render it highly practical for clinical applications, enabling healthcare professionals to prioritize treatments effectively.

## Data availability statement

The raw data supporting the conclusions of this article will be made available by the authors, without undue reservation.

## Ethics statement

The studies involving humans were approved by Ethics Committee of the First Affiliated Hospital of Chengdu Medical College. The studies were conducted in accordance with the local legislation and institutional requirements. The ethics committee/institutional review board waived the requirement of written informed consent for participation from the participants or the participants’ legal guardians/next of kin because this clinical study is a retrospective observational study, and the names of the participants will be blinded. The data obtained from the clinical study will only be used for the publication of this study’s paper. The patient information in the research report only uses the patient ID number and does not involve the patient's name or other information. Therefore, this clinical study does not pose a direct risk to the patient themselves.

## Author contributions

RD: Conceptualization, Formal analysis, Writing – original draft. YL: Writing – original draft. TF: Methodology, Software, Writing – review & editing. TR: Writing – review & editing.
