# Supplementary material for: Development of a nomogram for predicting malnutrition in elderly hospitalized cancer patients: a cross-sectional study in China
Source: Front Nutr. 2024 Jul 8;11:1396293. doi: 10.3389/fnut.2024.1396293 (PMC11260752; doi:10.3389/fnut.2024.1396293)
Supplement: Supplementary file 1 [file Data_Sheet_1.docx]

Title “Development of a Nomogram for Predicting Malnutrition in Elderly Hospitalized Cancer Patients: A Cross-Sectional Study in China

”

**Supplementary Materials**

**Supplementary Table Legend**

**Supplementary Table 1.** The coefficients of Lasso regression analysis

**Supplementary Table 2.** Results of Univariate Logistic regression

**Supplementary Table 3.** Classification accuracy for prediction at different risk cutoff points for the model in training cohort.

**Supplementary Table4.** Classification accuracy for prediction at different risk cutoff points for the model in internal test cohort.

**Supplementary Figure Legend**

**Supplementary Figure 1.** The ROC curve for predictions with a single independent variable.

Table 1:The coefficients of Lasso regression analysis

| Coefficient | variable |
| --- | --- |
| 1.94478281 | (Intercept) |
| 0.00000000 | Sex_level_2 |
| 0.03572094 | Age_level_ |
| 0.00000000 | Education _level_2 |
| 0.00000000 | Education _level_3 |
| 0.00000000 | Radiotherapy _level_1 |
| 0.00000000 | Chemotherapy _level_1 |
| 0.00000000 | Immunotherapy _level_1 |
| 0.00000000 | Pain Score _level_ |
| 0.00000000 | T_level_1 |
| 0.00000000 | T_level_2 |
| 0.00000000 | T_level_3 |
| 0.00000000 | T_level_4 |
| 0.00000000 | N_level_1 |
| 0.00000000 | N_level_2 |
| 0.00000000 | N_level_3 |
| 0.00000000 | N_level_4 |
| 0.00000000 | M_level_1 |
| 0.00000000 | M_level_4 |
| 0.00000000 | Smoke _level_1 |
| 0.00000000 | Diabetes _level_1 |
| 0.00000000 | Drink _level_1 |
| 0.00000000 | Hypertension _level_1 |
| -0.08237891 | BMI_level_ |
| 0.00000000 | Hemoglobin _level_ |
| 0.00000000 | Globulin _level_ |
| 0.00000000 | C-Reactive Protein _level_ |
| 0.00000000 | Neutrophils _level_ |
| -0.03178570 | ALB_level_ |
| 0.00000000 | Leukocyte _level_ |
| -0.02638686 | ADL_level_ |

BMI,body mass index;ALB, albumin ;ADL, activities of daily living.

Table 2:Results of Univariate Logistic regression

| **Characteristic** | **N** | **Event N** | **OR**^1^ | **95% CI**^1^ | **p-value** |
| --- | --- | --- | --- | --- | --- |
| Age | 315 | 147 | 1.15 | 1.10, 1.20 | <0.001 |
| BMI | 315 | 147 | 0.73 | 0.66, 0.79 | <0.001 |
| ALB | 315 | 147 | 0.87 | 0.83, 0.91 | <0.001 |
| ADL | 315 | 147 | 0.96 | 0.95, 0.96 | <0.001 |
| ^1^OR = Odds Ratio, CI = Confidence Interval | | | | | |

Table 3:Classification accuracy for prediction at different risk cutoff points for the model in training cohort

| Risk score threshold | Linear Predictor Cutoff Point | Sensitivity (%) | Specificity (%) | PPV (%) | NPV (%) | Accuracy (%) | Precision (%) | Recall (%) | F1 |
| --- | --- | --- | --- | --- | --- | --- | --- | --- | --- |
| ≥ 0% | -Inf | 100.0 | 0.0 | 46.7 |  | 46.7 | 46.7 | 100.0 | 0.636 |
| ≥ 10% | -2.1972246 | 100.0 | 51.2 | 64.2 | 100.0 | 74.0 | 64.2 | 100.0 | 0.782 |
| ≥ 20% | -1.3862944 | 99.3 | 73.2 | 76.4 | 99.2 | 85.4 | 76.4 | 99.3 | 0.864 |
| ≥ 30% | -0.8472979 | 95.2 | 80.4 | 80.9 | 95.1 | 87.3 | 80.9 | 95.2 | 0.875 |
| ≥ 40% | -0.4054651 | 91.2 | 84.5 | 83.8 | 91.6 | 87.6 | 83.8 | 91.2 | 0.873 |
| ≥ 50% | 0.0000000 | 86.4 | 85.1 | 83.6 | 87.7 | 85.7 | 83.6 | 86.4 | 0.849 |
| ≥ 60% | 0.4054651 | 81.0 | 85.1 | 82.6 | 83.6 | 83.2 | 82.6 | 81.0 | 0.818 |
| ≥ 70% | 0.8472979 | 66.0 | 87.5 | 82.2 | 74.6 | 77.5 | 82.2 | 66.0 | 0.732 |
| ≥ 80% | 1.3862944 | 54.4 | 89.9 | 82.5 | 69.3 | 73.3 | 82.5 | 54.4 | 0.656 |
| ≥ 90% | 2.1972246 | 27.9 | 94.6 | 82.0 | 60.0 | 63.5 | 82.0 | 27.9 | 0.416 |
| ≥ 100% | Inf | 0.0 | 100.0 |  | 53.3 | 53.3 |  | 0.0 |  |

Table 4:Classification accuracy for prediction at different risk cutoff points for the model in internal test cohort

| Risk score threshold | Linear Predictor Cutoff Point | Sensitivity (%) | Specificity (%) | PPV (%) | NPV (%) | Accuracy (%) | Precision (%) | Recall (%) | F1 |
| --- | --- | --- | --- | --- | --- | --- | --- | --- | --- |
| ≥ 0% | -Inf | 100.0 | 0.0 | 45.9 |  | 45.9 | 45.9 | 100.0 | 0.629 |
| ≥ 10% | -2.1972246 | 100.0 | 43.8 | 60.2 | 100.0 | 69.6 | 60.2 | 100.0 | 0.752 |
| ≥ 20% | -1.3862944 | 100.0 | 65.8 | 71.3 | 100.0 | 81.5 | 71.3 | 100.0 | 0.832 |
| ≥ 30% | -0.8472979 | 95.2 | 68.5 | 72.0 | 94.3 | 80.7 | 72.0 | 95.2 | 0.819 |
| ≥ 40% | -0.4054651 | 90.3 | 72.6 | 73.7 | 89.8 | 80.7 | 73.7 | 90.3 | 0.812 |
| ≥ 50% | 0.0000000 | 87.1 | 75.3 | 75.0 | 87.3 | 80.7 | 75.0 | 87.1 | 0.806 |
| ≥ 60% | 0.4054651 | 77.4 | 78.1 | 75.0 | 80.3 | 77.8 | 75.0 | 77.4 | 0.762 |
| ≥ 70% | 0.8472979 | 69.4 | 79.5 | 74.1 | 75.3 | 74.8 | 74.1 | 69.4 | 0.717 |
| ≥ 80% | 1.3862944 | 54.8 | 83.6 | 73.9 | 68.5 | 70.4 | 73.9 | 54.8 | 0.630 |
| ≥ 90% | 2.1972246 | 22.6 | 86.3 | 58.3 | 56.8 | 57.0 | 58.3 | 22.6 | 0.326 |
| ≥ 100% | Inf | 0.0 | 100.0 |  | 54.1 | 54.1 |  | 0.0 |  |


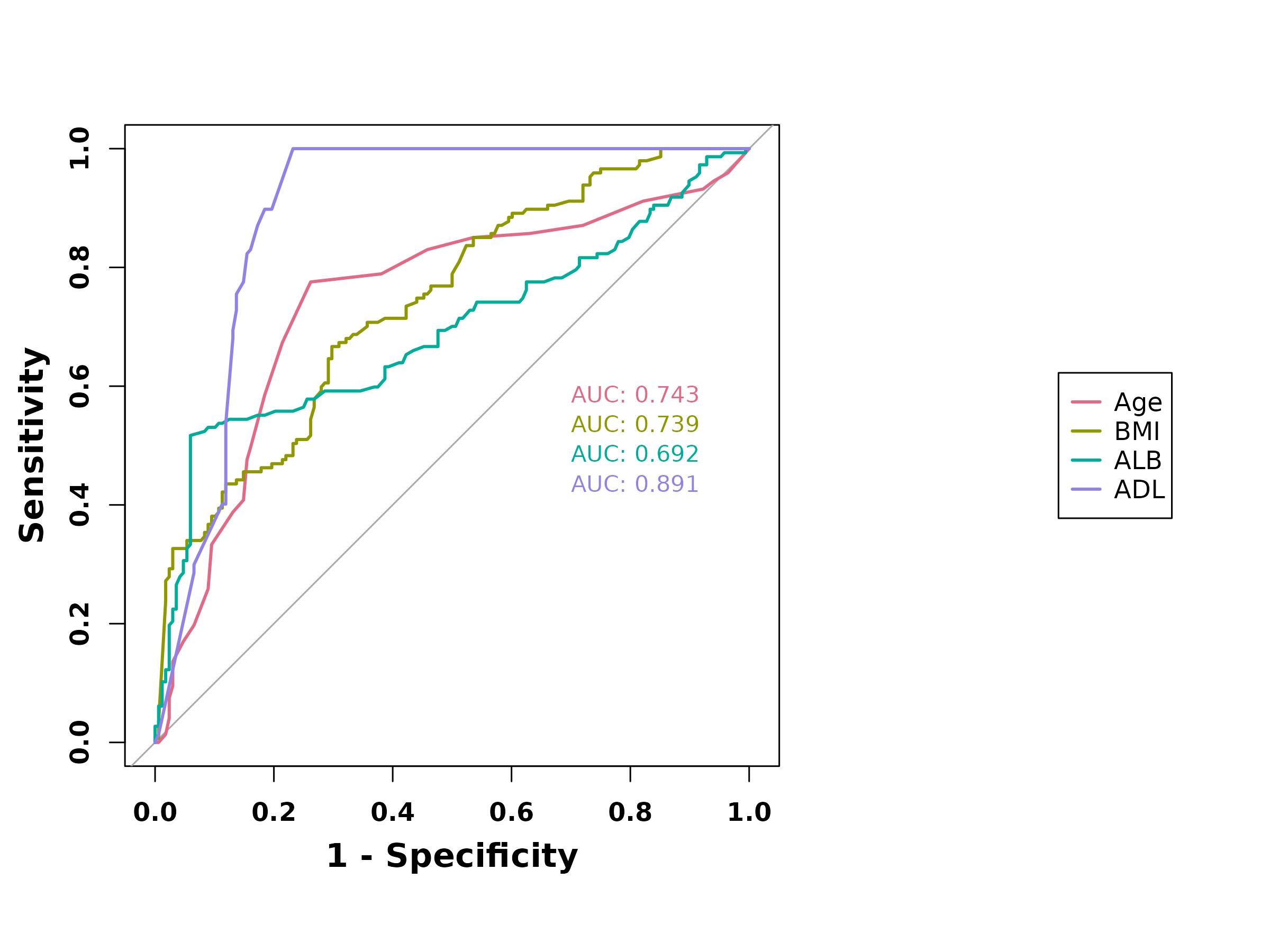


Figure 1：The ROC curve for predictions with a single independent variable.

BMI,body mass index;ALB, albumin ;ADL, activities of daily living.
